# An Attitude Prediction Method for Autonomous Recovery Operation of Unmanned Surface Vehicle

**DOI:** 10.3390/s20195662

**Published:** 2020-10-03

**Authors:** Yang Yang, Ping Pan, Xingang Jiang, Shuanghua Zheng, Yongjian Zhao, Yi Yang, Songyi Zhong, Yan Peng

**Affiliations:** School of Mechatronic Engineering and Automation, Shanghai University, Shanghai 200444, China; yangyang_shu@shu.edu.cn (Y.Y.); parkin@shu.edu.cn (P.P.); jackong@shu.edu.cn (X.J.); 18717927360@163.com (S.Z.); zhaoyongjian@shu.edu.cn (Y.Z.); zhongsongyi@shu.edu.cn (S.Z.); pengyan@shu.edu.cn (Y.P.)

**Keywords:** unmanned surface vehicle (USV), launch and recovery system (L&RS), attitude prediction, convolutional neural network (CNN), long short-term memory (LSTM) neural network

## Abstract

The development of launch and recovery technology is key for the application to the unmanned surface vehicle (USV). Also, a launch and recovery system (L&RS) based on a pneumatic ejection mechanism has been developed in our previous study. To improve the launch accuracy and reduce the influence of the sea waves, we propose a stacking model of one-dimensional convolutional neural network and long short-term memory neural network predicting the attitude of the USV. The data from experiments by “Jinghai VII” USV developed by Shanghai University, China, under levels 1–4 sea conditions are used to train and test the network. The results show that the stabilized platform with the proposed prediction method can keep the launching angle of the launching mechanism constant by regulating the pitching joint and rotation joint under the random influence from the wave. Finally, the efficiency and effectiveness of the L&RS are demonstrated by the successful application in actual environments.

## 1. Introduction

Many countries are developing intelligent unmanned maritime equipment for marine exploitation and protection of maritime rights and interests owing to the rich biological and mineral resources. Unmanned surface vehicle (USV) is a kind of unmanned surface platform with autonomous navigation and obstacle avoidance ability, and it can independently complete tasks such as marine environment information perception, inshore island mapping, and disaster rescue, which is suitable for dangerous and human-unsuited missions instead of manned surface boats vessels [1,2,3,4,5].

Limited by endurance, the USV is usually carried by the mother ship to the mission area and then placed on the surface for autonomous operation. After the mission is completed, it would be recovered to the mother ship’s deck. Therefore, the launch and recovery technology is a key technology for the application of USV [6]. The launch and recovery system (L&RS) for the manned surface boat can be divided into two types. The first one is the stern ramp type, which is mainly composed of a slide and a winch. Kern et al. have designed a device with an inclined chute and traction mechanism that can recover both autonomous underwater vehicle and remote-operated vehicle [7]. Hayashi et al. developed a set of devices composed of an obstacle avoidance system, slope L&RS, and a matching remote control for reducing the number of operators in the recovery process [8]. However, the requirement of slideway is limited to the application range, and thus, the davit system is more widely used compared with the slide system. The davit is generally installed on both sides of the mother ship’s deck. During the operation, the USV is out of the ship’s side and is lowered to or hoisted from the sea surface [9]. The RHP L&RS developed by Global davit gmbH can be used for the launch and recovery of boats with mass from 1000 to 3500 kg [10]. Marine Equipment Pellegrini, a company from Italy, has developed a marine L&RS capable of operating at level 6 sea conditions and has strong adaptability and large load capacity. The existing L&RS, however, requires human intervention to operate the boat, which is a difficulty when this is used for USV.

One of the challenges of the launch and recovery technology is the connection of USV to the mother ship’s recovery system, dealing with the uncertainty and randomness under the impact of the waves, especially during high seas conditions. Thus, predicting the movement trend of USVs is important during launch and recovery operations. Consequently, several methods have been used, such as statistical forecast, Kalman filter, and time series [11]. Wiener et al. proposed an optimal linear prediction method based on the minimum mean square error [12]. The method can obtain better prediction results just within 5–6 s; however, the prediction error significantly increased with the prolonged prediction time [13]. Furthermore, Dodin and Sidar obtained the ship’s motion state equation based on the force analysis and then deduced the multi-step prediction [14]. On the basis of the autoregressive model, Peng et al. proposed a real-time modeling and prediction method for predicting attitude motion of large ships under random wave action. The method is found suitable for application under non-stationary motion conditions, and the prediction time takes only 7–10 s [15]. Khan et al. combined the autoregressive model and the moving average model with an artificial neural network for predicting ship motion to achieve better prediction accuracy [16]. On the other hand, methods of attitude prediction were focused on large tonnage ships. The amplitude and frequency of the attitude are smaller than that of common USVs because of the inertia. In improving the maneuverability, the mass of the USV should be generally small. However, because of the influence of the wind and waves, large and high-frequency changes in attitudes have occurred.

For previously developed L&RS based on the pneumatic ejection mechanism, this study presents the attitude prediction for USV to improve the operation success rate. The rest of the paper is organized as follows. Section 2 introduces the concept and mechanism of the L&RS. In Section 3, the USV attitude prediction algorithm for stacking one-dimensional convolutional neural network (1D CNN) and long short-term memory (LSTM) neural network is proposed for improving the aiming accuracy of the L&RS under the influence of the waves. The experiments in Section 4 verify the validity of the algorithm, and efficiency of the automatic L&RS. The conclusions and plans for future studies are presented in Section 5.

## 2. Launch and Recovery System

### 2.1. Mechanism

**Figure 1:**
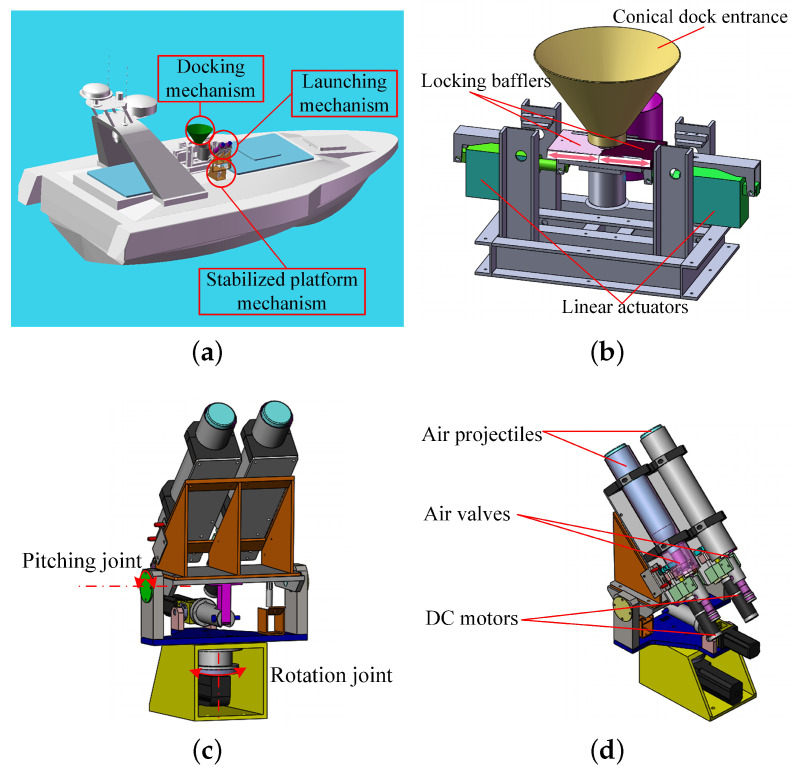
Schematic of an automated L&RS. (**a**) Concept. (**b**) Docking mechanism. (**c**) Stabilized platform mechanism. (**d**) Launching mechanism.

A L&RS for USV based on pneumatic projectile has been developed in our previous study [17]. As shown in Figure 1, it is composed of a launching mechanism, a 2-degree-of-freedom-stabilized platform mechanism and a docking mechanism. During the launching process, the USV was lowered to the sea surface by the davit, and then the locking mechanism separated the USV from the conical butt joint.

**Figure 2:**
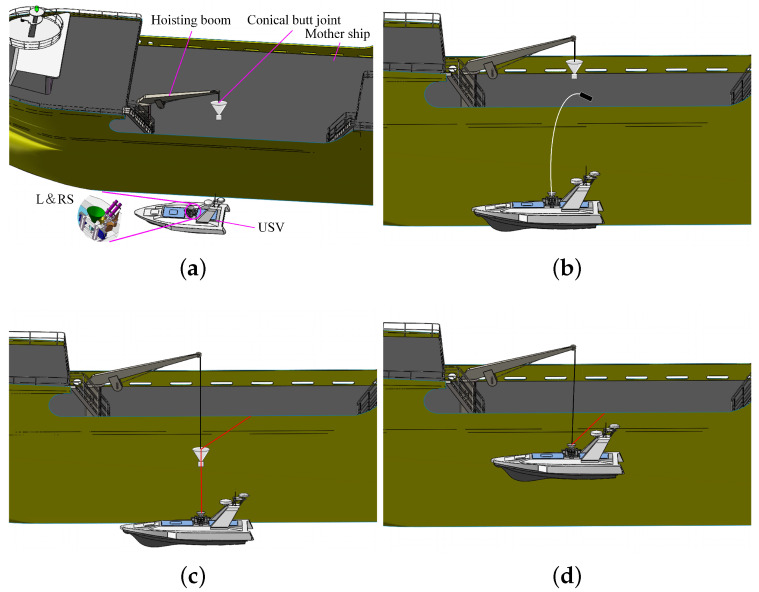
Operation process in recovering a USV. (**a**) Homing and aiming phase. (**b**) Launching phase. (**c**) Docking phase. (**d**) Lifting phase.

The recovery process after the completion of a mission is as follows (Figure 2).

1. First, by automatic regulation of the pitching and rotation joints of the stabilized platform mechanism, the launching mechanism is aimed to the mother ship’s deck (Figure 2a).
2. After the launching switch was acted, the air projectile was separated from the catapult mechanism and was driven by the high-pressure gas. It drives the guide rope to drop on the mother ship’s deck (Figure 2b).
3. Then, the mother ship’s crew passes the guide rope through the hole of the conical butt joint, and the conical butt joint slides along with the guide rope into the docking mechanism (Figure 2c).
4. Finally, the docking mechanism locks the conical butt joint, and the USV is lifted from the sea surface by the davit (Figure 2d).

### 2.2. Launching Angle

The accurate launching of the air projectile to the mother ship is important for the recovery operation of USV. When the launching angle is too large, it could result in high elevation and short-range, causing the air projectile to fall into the sea. Hence, when the launching angle is too small, it could result in low elevation and longer range, causing the air projectile to hit the mother ship’s sidewall. Therefore, during the recovery process, it is required to regulate the launching angle of the stabilized platform according to the measured distance and direction information between the USV and the mother ship.

**Figure 3:**
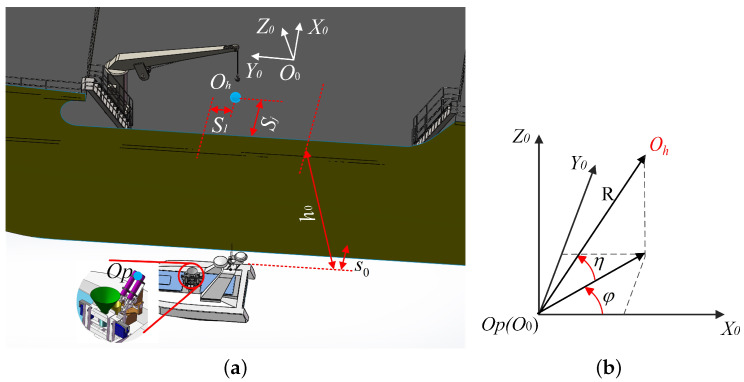
Coordinate systems. (**a**) Overview. (**b**) The azimuth angle φ and the elevation angle η in the world coordinate system.

A world coordinate system $O_{0}X_{0}Y_{0}Z_{0}$ is established, as shown in Figure 3a. The $O_{0}Y_{0}$ axis points to the bow; the $O_{0}Z_{0}$ axis is perpendicular to the sea level, opposite to the direction of gravity, and direction of the $O_{0}X_{0}$ is determined by the right-hand rule. The coordinates of the port of the canister launcher $O_{p}$ and the target landing point of the air projectile $O_{h}$ are denoted by ($x_{p}$,$y_{p}$,$z_{p}$) and ($x_{h}$,$y_{h}$,$z_{h}$), respectively. On the basis of the aerodynamics, the desired angle of the azimuth angle φ and the elevation angle η in the world coordinate system (Figure 3b) can be calculated as follows [18]:

(1) $$\varphi =\arctan\frac{y_{h}-y_{p}}{x_{h}-x_{p}}=\arctan\frac{\mathrm{sgn}\left(x_{h}-x_{p}\right)\cdot s_{1}}{s_{0}+s_{j}}$$

(2) $$\eta =\arcsin\left[\frac{cW(\frac{c^{2}-1}{e}e^{\frac{b^{2}l_{h}}{m^{2}g}})}{c^{2}-1-W\left(\frac{c^{2}-1}{e}e^{\frac{b^{2}l_{h}}{m^{2}g}}\right)}\right]$$

(3) $$c=\frac{bv_{0}}{mg}$$

where $s_{0}$ is the distance between the USV and mother ship’s side, $s_{1}$ is the distance between the USV and the landing point along the direction of the $O_{0}Y_{0}$ axis, $h_{0}$ is the altitude difference between the mother ship and the USV, $s_{j}$ is the distance from the mother ship’s side to the landing point, $l_{h}$ and $v_{0}$ are the elevation and initial velocity of the air projectile, respectively, *m* is the mass of air projectile, and *b* is the damping coefficient.“sgn” is a symbolic function, which returns an integer variable indicating the sign of the argument:

(4) $$sgn\left(x\right)=\left\{\begin{array}{cc} 1, & x>0\\ 0, & x=0\\ -1, & x<0 \end{array}\right.$$

The Lambert *W* function is a multivalued complex function:

(5) $$W\left(x\right)e^{W(x)}=x$$

where *x* is a complex number. The detailed relationship between *x* and the Lambert W(x) function can be found in [19].

By coordinate transformation, the corresponding angles of the pitching and rotation joints of the stabilized platform can be derived. Thus, the launching angle can be kept constant by regulating the stabilized platform in real time to compensate for the attitude change of the USV due to the influence of sea waves.

## 3. USV Attitude Prediction

Having the features of being small in size and lightweight, the USV has superior maneuverability over the common manned surface vehicle. However, the changes in attitude due to the influence of waves will affect the landing point accuracy of air projectile in a recovery operation. By reducing the tracking error of the catapult mechanism due to time delays, we predict the attitude of the USV based on the previous state information. In this study, the LSTM neural network model is used to predict the attitude of the USV at sea in real time. To improve the performance of the prediction model, 1D CNN is superimposed on the LSTM. It can reduce the fluctuation range of prediction results and prediction errors, resulting in a successful recovery operation.

### 3.1. LSTM Neural Network

LSTM is an advanced recurrent neural network (RNN) structure that can learn and predict time series data [20,21]. For an ordinary RNN network, the output at the time *t* is as follows:

(6) $$Y_{t}=\delta (W_{o}X_{t}+U_{o}S_{t}+b_{o})$$

where $X_{t}$ is the input at the current moment; $S_{t}$ is the state of the network at time *t*, which is derived from the output of the network at the previous moment (i.e., $S_{t}=Y_{t-1}$); $W_{o}$ is the weight matrix of the input; $U_{o}$ is the weight matrix for the states; $b_{o}$ is the bias; and δ is the activation function of the network. After $Y_{t}$ is inputted to the softmax layer, the final prediction result can be obtained as follows:

(7) $$prediction=softmax(V_{s}Y_{t}+b_{s})$$

where $V_{s}$ and $b_{s}$ are the weights of the softmax layer. Because state $S_{t}$ is a recursive variable, the derivative term increases with the time step when calculating the gradient, causing the gradient to disappear. To solve this problem, we improved the LSTM neural network on the basis of the original RNN network.

**Figure 4:**
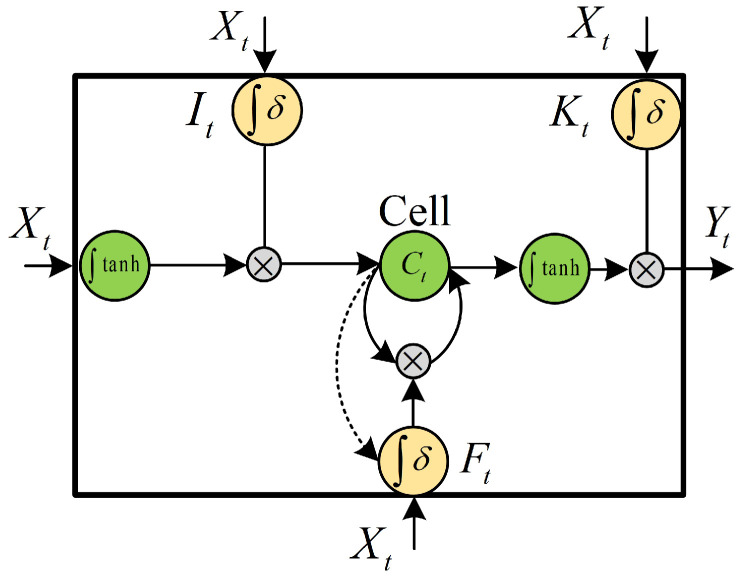
The internal structure of the LSTM neurons.

Figure 4 shows the internal structure of the LSTM, which adds a memory cell state $C_{t}$ on the original basis. During the training process, the signal is not only controlled by the input and output but also passes the forgetting control unit. The forget gate $F_{t}$ enables the network to delete some memory cell state information according to the previous training feedback without changing the weight, and it also selects certain neurons to update the weight. Also, an input gate $I_{t}$ and an output gate $K_{t}$ are added to make the model nonlinear. The input gate determines the amount of current input information used to calculate the carrying value. The output gate determines the amount of output from the carrying value to the final state. The state information of the three gates is calculated as follows:

(8) $$\left\{\begin{array}{c} I_{t}=\delta_{i}\left(W_{i}X_{t}+U_{i}S_{t}+b_{i}\right)\\ F_{t}=\delta_{f}\left(W_{f}X_{t}+U_{f}S_{t}+b_{f}\right)\\ K_{t}=\delta_{k}\left(W_{k}X_{t}+U_{k}S_{t}+b_{k}\right) \end{array}\right.$$

The memory cell state information carried by the network at the next moment is as follows:

(9) $${\tilde{C}}_{t}=\tanh(W_{c}X_{t}+U_{c}S_{t}+b_{c})$$

(10) $$C_{t}=F_{t}*C_{t-1}+I_{t}*{\tilde{C}}_{t}$$

where ${\tilde{C}}_{t}$ is the candidate value used to calculate the memory cell state information. The output of the network is as follows:

(11) $$Y_{t}=K_{t}*\tanh(C_{t})$$

where $Y_{t}$ is the input to the softmax layer, obtaining the final prediction result. The carrying information can still be retained even after several time steps. The final output can derive long-term dependencies from the carrying information, thus solving the problem of gradient disappearance. At the same time, the input gate and the output gate can also adjust the influence of the output of different timing on the model, so it can effectively solve the problem of the gradient explosion of RNN.

### 3.2. One-Dimensional Convolutional Neural Network

Although LSTM has a suitable performance for processing time series, it is difficult to apply to a huge number of input data, which significantly reduced the calculation efficiency. 1D CNN is an effective method in dealing with sequence objects. It can extract high-level features from local input data through convolution operations, which can efficiently use data and reduce the input dimension. As a result, computational cost can be significantly reduced [22].

**Figure 5:**
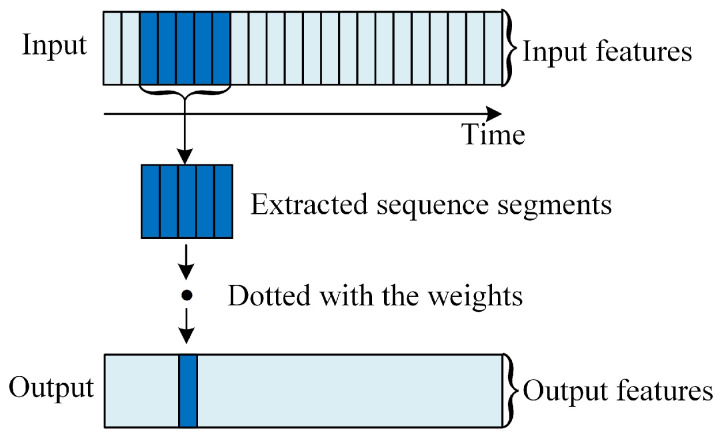
The working principle of 1D CNN. Each output time step is obtained using a small segment of the input sequence in the time dimension.

As shown in Figure 5, a local one-dimensional sequence segment is extracted from the original sequence in a 1D CNN. It is dotted with the weights in the convolution kernel to generate a shorter one-dimensional sequence. The sequence is trained as the input to the LSTM layer. As the input sequence length is shortened, the input dimension of the LSTM layer and the required training parameters are reduced. Therefore, the computing load can be effectively reduced, and the training time of the network can be shortened. Also, the 1D CNN extracts more advanced and abstract features from the original sequence in advance, so that the data use is high and the performance of the network can be effectively improved.

### 3.3. USV Attitude Data

Owing to the strong correlation between the attitude motion sequences of USV, the attitude in the next moment can be predicted by the LSTM neural network based on the attitude sequence value within the past time. In this paper, the training data are measured by a six-axis gyroscope mounted on the “Jinghai VII” USV developed by Shanghai University. The attitude data include posture angle and angular velocity in the heading, pitch, and roll directions of the USV. The measurement frequency of the sensing system is 10 Hz, which is far faster than that of the large tonnage ships. The experimental data with a time step of 0.1 s can be converted into dimensionless data through standardized formulas:

(12) $$x*\left(t\right)=\frac{x\left(t\right)-x_{min}\left(t\right)}{x_{max}\left(t\right)-x_{min}\left(t\right)}$$

where *t* is time; x(t) is input data at time *t*; $x_{max}\left(t\right)$ and $x_{min}\left(t\right)$ are maximum and minimum values of the input data at time *t*, respectively; and x*(t) is the normalized value of the input data in which the range is from 0 to 1.

### 3.4. Determination of Hyper-Parameters

In this study, we applied a neural network model with two hidden layers and ten hidden units. The input layer inputs the attitude angle and angular velocity information of the USV in one direction. The output layer uses the tanh function as the activation function to output the predicted result. The uniform distribution randomly generates the network weights. The range is (−limit, limit):

(13) $$limit=\sqrt{\frac{6}{n_{j}+n_{j+1}}}$$

where $n_{j}$ is the number of units in the layer *j* network. The bias is initialized at 0. The square sum of the difference between the predicted value and the actual value of the output is selected as the loss function. By minimizing the loss function, all weights and bias parameters in the network can be obtained:

(14) $$loss=\frac{\sum_{j=1}^{n}{\left(y_{i}-{y_{i}}^{p}\right)}^{2}}{n}$$

where $y_{i}$ is the actual value, ${y_{i}}^{p}$ is the predicted value, and *n* is the total number of data.

The loss function can also be used to evaluate the accuracy of the neural network training model. The trained heading, pitch, and roll neural network models of the USV can be respectively derived from the posture angle and angular velocity in three directions.

### 3.5. Training Process

**Figure 6:**
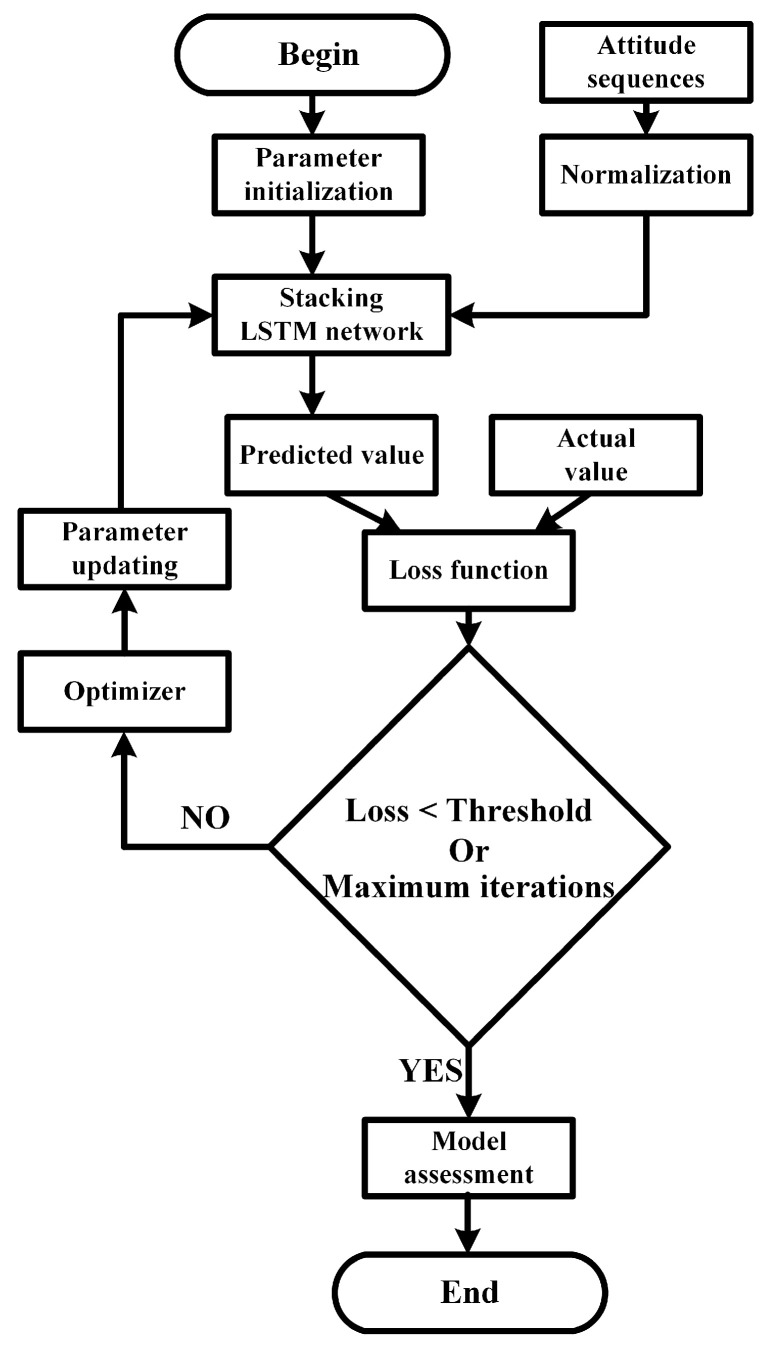
The training process of the 1D CNN-LSTM neural network.

As shown in Figure 6, first, the weights and biases of the stacked LSTM network are initialized. After entering the normalized attitude data, the forecasted value of heading, roll, and pitch angles can be obtained. Equation (14) is used to calculate the loss between the desired value and the real value. Finally, the gradient descent method is used to constantly adjust the weight and bias until the loss value converges or the number of iterations reaches the peak.

It is noted that a large size of database takes a long training time. Thus, a reasonable batch size needs to be set to reduce the number of iterations required for the training model. To obtain the suitable batch size, it was set to 1 at the beginning, and then the value increased until the improvement in training accuracy was no longer apparent in this study.

## 4. Experiments and Discussion

### 4.1. Experimental Overview

To verify the validity of the proposed prediction algorithm, we used the experimental data measured from the “Jinghai VII” USV under levels 1–4 sea conditions for training the testing. Table 1 lists the specifications of the USV. According to [23,24], the sea level conditions were generally defined by the ranges of significant wave heights as listed in Table 2. The data set contains the posture angles and angular velocities of the USV in the heading, pitch, and roll directions. Each set has approximately 3000 sets of data, in which 60%, 20%, and 20% were taken as training, verification, and test sets, respectively. The batch size is 128 in both training process and validation process.

To prevent model overfitting, we took samples 100 times per turn for a total of 50 rounds. The neural network model is trained by inputting the angle and angular velocity information simultaneously. To demonstrate the performance of the proposed method, we compared the results with that predicted by the original LSTM, Nonlinear Autoregressive Exogenous Model (NARX) network, and Time Delay Neural Network (TDNN) under 1–4 sea levels.

### 4.2. Results and Discussion

**Figure 7:**
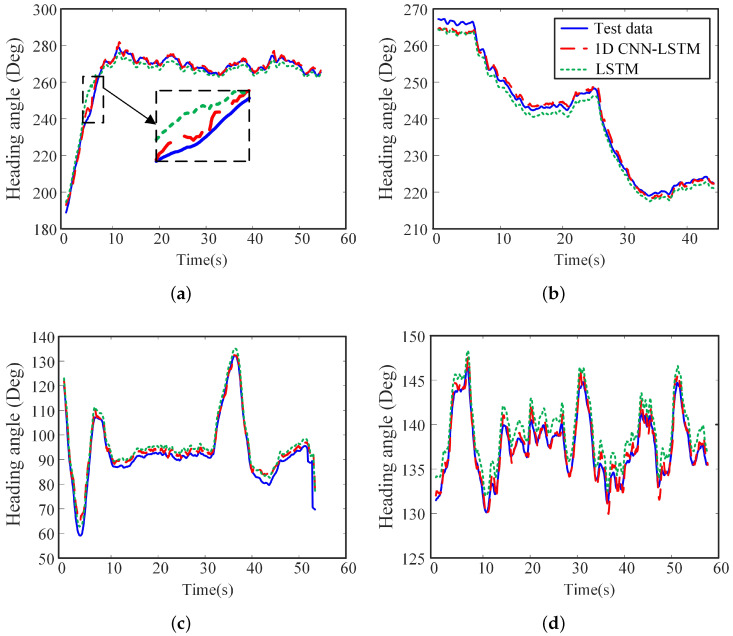
Comparison of the predict results and actual results by LSTM with CNN and LSTM without CNN at different sea level. (**a**) Level 1 sea condition. (**b**) Level 2 sea condition. (**c**) Level 3 sea condition. (**d**) Level 4 sea condition.

To evaluate the performance of the 1D CNN-LSTM neural network, we compared the effect of that LSTM neural network model, in which the parameter settings and training test data are the same for the two models. The results of the heading angle of USV are taken as an example. As shown in Figure 7a, although the sea condition is at level 1, the heading angle fluctuates greatly because of the slight weight of the USV. Both neural network models can predict the trend of the USV attitude, but the predicted results by LSTM neural network have a rougher degree of agreement with the actual curve. It is deduced that as the data set is small, the original LSTM neural network is difficult to learn the changing characteristics of the heading angle, reducing the accuracy of the prediction model. It can be seen from the enlarged area of Figure 7a that when the heading angle suddenly changed, the prediction error of the LSTM neural network increased. However, the predicted results by 1D CNN-LSTM neural network can achieve higher accuracy. It is attributed that after the addition of CNN, the model can effectively extract the features of the USV attitude data and reduce the redundant information input to the LSTM neural network. CNN enables LSTM neural network to mine the deeper features of heading angle variation so that the predicted results are closer to the actual test data. Table 3 shows the comparison of the training speed and various losses of the model. It can be confirmed that the training speed of the LSTM neural network is 22 ms/step, whereas the training speed of the 1D CNN-LSTM network model is increased by 55% to 10 ms/step. The LSTM test loss was 0.0511, whereas the test loss of the 1D CNN-LSTM network model was reduced by 59% to 0.0212. The 1D CNN-LSTM neural network has higher training efficiency and higher prediction accuracy.

The proposed prediction algorithm is required to guarantee suitable prediction accuracy for various sea conditions and environments. To demonstrate the performance of the proposed network model under unknown complex sea conditions and higher noise disturbances, we showed the results of the heading angle under level 2–4 sea conditions in Figure 7b–d, respectively. As shown in Table 3, the test error of the LSTM neural network increased with the increase of sea level. It means that the complexity of high sea conditions has a great influence on the accuracy of the LSTM neural network prediction result. However, the predicted results by the 1D CNN-LSTM network model after training agreed with the test data well. The proposed network model test error is approximately 0.015–0.025.

**Figure 8:**
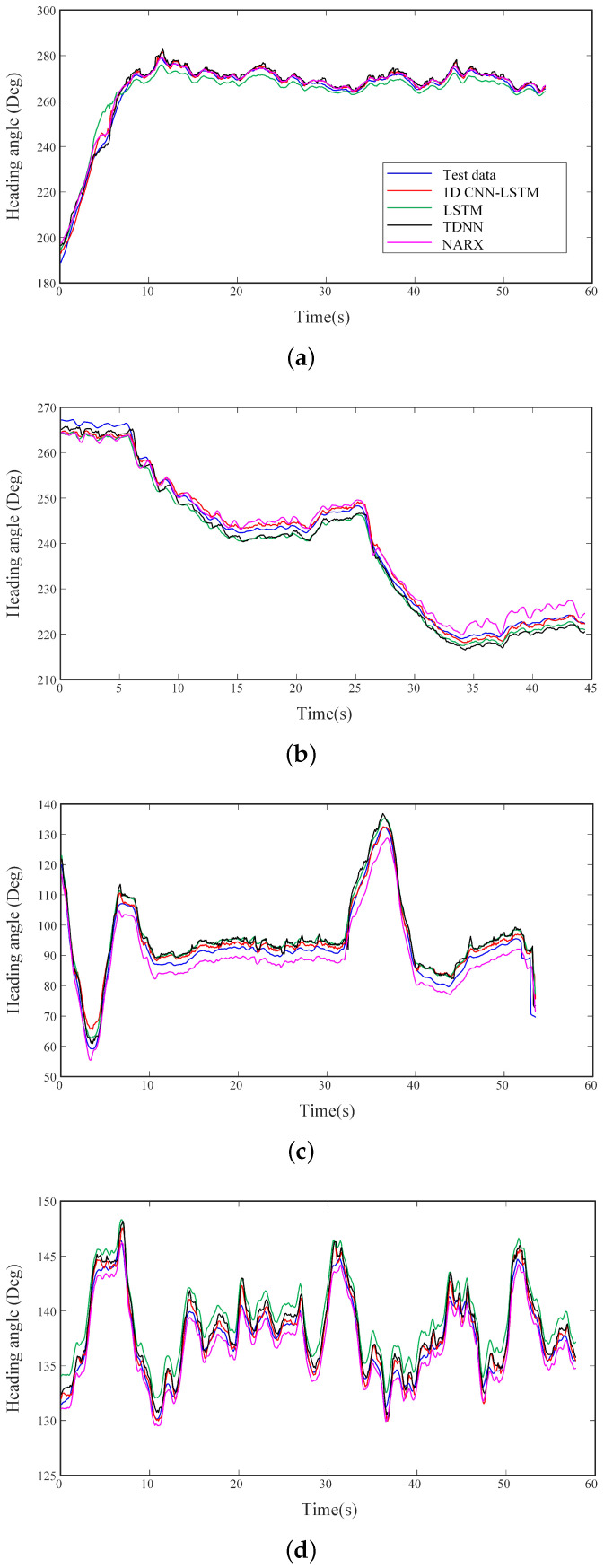
Comparison of the heading angle by 1D CNN-LSTM network, LSTM network, NARX network and TDNN network at different sea level. (**a**) Level 1 sea condition. (**b**) Level 2 sea condition. (**c**) Level 3 sea condition. (**d**) Level 4 sea condition.

As shown in Figure 8, all the neural network models can predict the trend of the USV attitude, in which the 1D CNN-LSTM can achieve the higher prediction accuracy than the other 3 networks. Table 3 demonstrated the comparison of the training speed and various losses of the four networks at different sea level. Compared with NARX, the training speed of 1D CNN-LSTM increased by 25% (level 1), 18% (level 2), 17% (level 3) and 21% (level 4), and test loss decreased by 4% (level 1), 43% (level 2), 16% (level 3) and 6% (level 4). Although the training speed of 1D CNN-LSTM has slight increase with TDNN, the test loss was 25% (level 1), 39% (level 2), 29% (level 3) and 26% (level 4) lower than that of TDNN. In addition, it can be found that the training speed of 1D CNN-LSTM in levels 1–4 sea conditions is increased by 45%, 30%, 25% and 29% respectively, compared with the test loss of LSTM. The test loss of 1D CNN-LSTM in levels 1–4 sea conditions is reduced by 59%, 45%, 30%, and 65%, respectively, compared with the test loss of LSTM. It is noticed that the degree of the improvement in the prediction accuracy has no relationship with the sea level conditions.

It is noticed that compared with the common online prediction algorithms, 1D CNN-LSTM requires no calculation of the model parameters in real time. It ensures the high speed, real-time and reliability of the usv’s attitude data prediction. The fluctuation range of model prediction error under sea conditions is relatively stable regardless of the time, and the prediction error keep stable in the prolonged prediction time. Therefore, the 1D CNN-LSTM network is superior to the other three networks in terms of prediction accuracy, adaptability, and efficiency. It can achieve suitable prediction accuracy under various sea conditions, which is important for the recovery operation of the USV.

### 4.3. Field Application

The autonomous launch and recovery operation of “Jinghai VII” USV was performed in the East China Sea to demonstrate the effect of the proposed attitude prediction method in the practical application. After the mission was completed, the USV returned to the vicinity of the mother ship in preparation for recovery. The mother ship was stopped, and the position was continuously changing due to the influence of sea waves. By the guidance of the navigation system, the USV finally stopped approximately 3 m away from the port side of the mother ship with the same heading.

After the propeller was stalled, the USV switched to the aiming phase. Owing to the influence of the wave, the attitude of the USV continuously changes. From Equations (1) and (2), the desired launching angle of the air projectile can be calculated from a distance between the stabilized platform and the target landing point in the world coordinate system measured by the laser rangefinder and GPS positioning system mounted on the USV.

**Figure 9:**
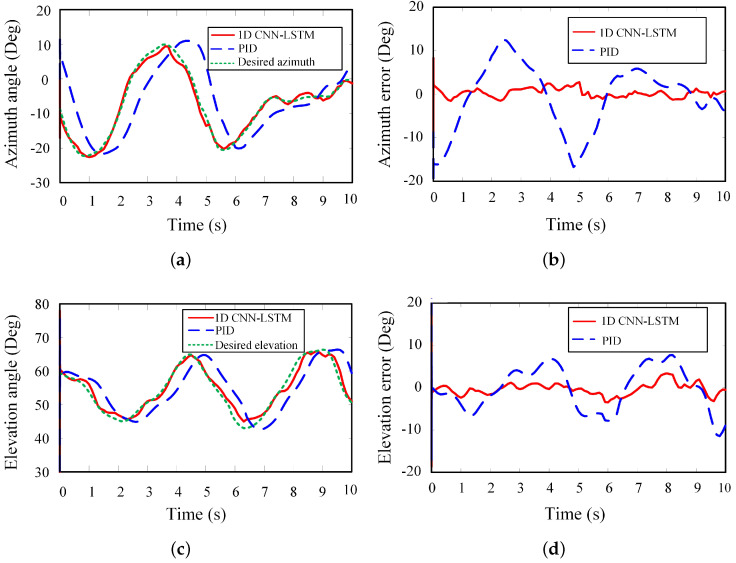
Comparison of the predict results and actual results in the field application. (**a**) Azimuth angle. (**b**) Error of azimuth angle. (**c**) Elevation angle. (**d**) Error of elevation angle.

Figure 9 demonstrates the effect of the proposed prediction algorithm on the stability control of the stabilized platform. It can be seen that the tracking error of the azimuth and elevation angles can be improved by using the proposed prediction algorithm. Without the attitude prediction, the stabilized platform regulated the azimuth and elevation angles based on the tracking error by the PID algorithm. As shown in Figure 9a,c, although the desired values and actual values have similar trends, it has the obvious time delay. It is attributed that the attitude of the USV changes instantly, and the launching angle of the air projectile changes synchronously with the change of the USV attitude. As the stabilized platform moves to the desired angle according to the calculated value at the previous moment, the USV attitude has changed. However, using the proposed prediction algorithm, the joint angles can follow the desired values with a slight tracking error. On the basis of the predicted results, the stabilized platform can respond in advance. It compensated the influence of the wave and increased the accuracy of the air projectile launch.

**Figure 10:**
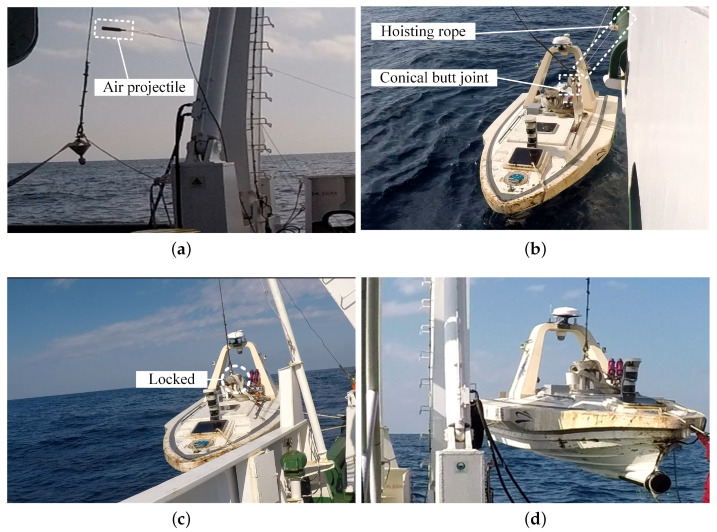
From (**a**–**d**), recovery process in field application. The recovery process took approximately 23 min. The efficiency of the aiming and launching of L&RS is suitable.

By applying the proposed prediction method, we can obtain an accurate projectile on the mother ship’s deck. As shown in Figure 10, following the operation process in Section 2.1, the mother ship’s crew operated the hoisting boom to drop the conical butt joint to slide into the conical dock entrance along with the guide rope. Then, the locking mechanism locked the conical butt joint, and the USV was lifted and placed on the mother ship’s deck.

During the operation, reducing the sway of the USV was time-consuming. Moreover, in reducing the sway of the USV during the lifting process, the operation required eight operators, a crane operator, a commander, and other people that could pull the USV in the head and stern directions through ropes. All people were working on the mother ship’s deck, and no operator is required onboard the USV. It significantly improved the safety of the USV recovery operation.

## 5. Conclusions and Future Work

To improve the launching accuracy of the developed pneumatic ejection mechanism-based L&RS of USV, we proposed a stacking model of 1D CNN and LSTM network in this study to predict the attitude of the USV. On the basis of the predicted results, the pitching joint and rotation joint of the stabilized platform can be regulated in real time to compensate for the disturbance of the waves, keeping the launching angle stable. The data from the experiments by “Jinghai VII” USV were used to train and test the proposed network. The results demonstrated that the algorithm has suitable prediction accuracy and calculation efficiency. From the filed application, it can be confirmed that the L&RS with the trained network can successfully recover USV. It requires no operator working on the USV, and it significantly improved safety and adaptability. In the future, the proposed method will be applied to model predictive control techniques, automatic speech recognition, etc. 

## Figures and Tables

**Table 1:** Specifications of “Jinghai VII” USV.

Parameters	Values	Unit
Length	8.2	m
Width	2.45	m
Height	1.84	m
Mass	3000	kg
Depth of immersion	0.5	m

**Table 2:** Definition of sea level conditions [23,24].

Sea Condition Level	Sea States	Significant Wave Height (m)
0	Calm (glassy)	0
1	Calm (ripples)	0–0.1
2	Smooth (wavelets)	0.1–0.5
3	Slight	0.5–1.25
4	Moderate	1.25–2.5
5	Rough	2.5–4.0
6	Very rough	4.0–6.0
7	High	6.0–9.0
8	Very high	9.0–14.0
9	Phenomenal (Extreme)	Over 14.0

**Table 3:** Comparison of the results by LSTM, 1D CNN-LSTM, NARX and TDNN.

Sea Condition	Network Model	Training Speed (ms/step)	Training Loss	Validation Loss	Test Loss
Level 1	LSTM	22	0.1013	0.1232	0.0511
	1D CNN-LSTM	12	0.0289	0.0128	0.0212
	NARX	16	0.0301	0.0120	0.0222
	TDNN	13	0.0247	0.0416	0.0283
Level 2	LSTM	20	0.0402	0.0264	0.0240
	1D CNN-LSTM	14	0.0284	0.0201	0.0132
	NARX	17	0.0308	0.0244	0.0232
	TDNN	14	0.0222	0.0188	0.0216
Level 3	LSTM	20	0.0309	0.0107	0.0310
	1D CNN-LSTM	15	0.0279	0.0211	0.0218
	NARX	18	0.0305	0.0186	0.0261
	TDNN	14	0.0212	0.0221	0.0308
Level 4	LSTM	21	0.0765	0.0461	0.0381
	1D CNN-LSTM	15	0.0415	0.0125	0.0133
	NARX	19	0.0377	0.0239	0.0141
	TDNN	16	0.0286	0.0232	0.0179

## References

[1] Furfaro T.C., Dusek J., Von Ellenrieder K.D. Design, construction, and initial testing of an autonomous surface vehicle for riverine and coastal reconnaissance. Proceedings of the OCEANS 2009.

[2] Breivik M., Hovstein V.E., Fossen T.I. (2008). Straight-Line target tracking for unmanned surface vehicles. Model. Identif. Control.

[3] Liu Z., Zhang Y., Yu X., Yuan C. (2016). Unmanned surface vehicles: An overview of developments and challenges. Annu. Rev. Control.

[4] Yang W., Chen C., Hsu C., Tseng C., Yang W. (2011). Multifunctional inshore survey platform with unmanned surface vehicles. Int. J. Autom. Smart Technol..

[5] Sonnenburg C., Woolsey C.A. (2013). Modeling, identification, and control of an unmanned surface vehicle. J. Field Robot..

[6] Fisher N., Gilbert G.R. (2016). Unmanned systems in support of future medical operations in dense urban environments. J. Article.

[7] Kern F.R. (2009). Launch and Recovery Devices for Water Vehicles and Methods of Use. U.S. Patent.

[8] Hayashi E., Kimura H., Tam C., Ferguson J., Laframboise J., Miller G., Kaminski C., Johnson A. Customizing an autonomous underwater vehicle and developing a launch and recovery system. Proceedings of the 2013 IEEE International Underwater Technology Symposium (UT).

[9] Wu G.X., Xie Y., Sun H.b., Zou J. (2009). Modeling and simulation of capsizing automatic recovery system for unmanned surface vehicle. J. Syst. Simul..

[10] Bergmann H.D. (2010). Device for a Watercraft for Picking up and Launching Boats. U.S. Patent.

[11] Peng X., Zhao X., Xu L. (2006). Real-time prediction algorithm research of ship attitude motion based on order selection with corner condition. Proceedings of the 2006 1st International Symposium on Systems and Control in Aerospace and Astronautics.

[12] Norbert W. (1949). Interpolation and Smoothing of Stationary Time Series.

[13] Bates M.R., Bock D., Powell F. (1957). Analog computer applications in predictor design. IRE Trans. Electron. Comput..

[14] Sidar M., Doolin B. (1983). On the feasibility of real-time prediction of aircraft carrier motion at sea. IEEE Trans. Autom. Control.

[15] Peng X.Y., Zhao X.R., Gao Q.F. (2007). Research on real-time prediction algorithm of ship attitude motion. J. Syst. Simul..

[16] Khan A., Bil C., Marion K., Crozier M. Real time prediction of ship motions and attitudes using advanced prediction techniques. Proceedings of the Congress of the International Council of the Aeronautical Sciences.

[17] Zheng S., Yang Y., Peng Y., Cui J., Chen J., Jiang X., Feng Y. (2019). An automated launch and recovery system for USVs based on the pneumatic ejection mechanism. Proceedings of the International Conference on Intelligent Robotics and Applications.

[18] Qian X., Yin Y., Zhang X., Li Y. (2016). Influence of irregular disturbance of sea wave on ship motion. J. Traff. Transp. Eng. China.

[19] Hu H., Zhao Y., Guo Y., Zheng M. (2012). Analysis of linear resisted projectile motion using the Lambert W function. Acta Mech..

[20] Ma C., Wang A., Chen G., Xu C. (2018). Hand joints-based gesture recognition for noisy dataset using nested interval unscented Kalman filter with LSTM network. Vis. Comput..

[21] Yang B., Sun S., Li J., Lin X., Tian Y. (2019). Traffic flow prediction using LSTM with feature enhancement. Neurocomputing.

[22] Pan H., He X., Tang S., Meng F. (2018). An improved bearing fault diagnosis method using one-dimensional CNN and LSTM. J. Mech. Eng.

[23] Nguyen T.D., Sørensen A.J., Quek S.T. (2007). Design of hybrid controller for dynamic positioning from calm to extreme sea conditions. Automatica.

[24] Price W.G. (1974). Probabilistic Theory of Ship Dynamics.

